# Optimization and Application of a Multiplex Digital PCR Assay for the Detection of SARS-CoV-2 Variants of Concern in Belgian Influent Wastewater

**DOI:** 10.3390/v14030610

**Published:** 2022-03-15

**Authors:** Tim Boogaerts, Siel Van den Bogaert, Laura A. E. Van Poelvoorde, Diala El Masri, Naomi De Roeck, Nancy H. C. Roosens, Marie Lesenfants, Lies Lahousse, Koenraad Van Hoorde, Alexander L. N. van Nuijs, Peter Delputte

**Affiliations:** 1 Toxicological Centre, University of Antwerp, Universiteitsplein 1, 2610 Antwerp, Belgium; alexander.vannuijs@uantwerpen.be; 2 Laboratory for Microbiology, Parasitology and Hygiene, University of Antwerp, Universiteitsplein 1, 2610 Antwerp, Belgium; diala.elmasri@uantwerpen.be (D.E.M.); naomi.deroeck@uantwerpen.be (N.D.R.); peter.delputte@uantwerpen.be (P.D.); 3 Scientific Directorate of Biological Health Risks, Service Transerversal Activities in Applied Genomics, Sciensano, J. Wytsmanstraat 14, 1050 Brussels, Belgium; laura.vanpoelvoorde@sciensano.be (L.A.E.V.P.); nancy.roosens@sciensano.be (N.H.C.R.); 4 Scientific Directorate of Epidemiology and Public Health, Service Epidemiology of Infectious Diseases, Sciensano, J. Wytsmanstraat 14, 1050 Brussels, Belgium; marie.lesenfants@sciensano.be; 5 Department of Bioanalysis, Ghent University, Ottergemsesteenweg 460, 9000 Ghent, Belgium; lies.lahousse@ugent.be; 6 Scientific Directorate of Infectious Diseases in Humans, Service Foodborne Pathogens, Sciensano, J. Wytsmanstraat 14, 1050 Brussels, Belgium; koenraad.vanhoorde@sciensano.be

**Keywords:** wastewater-based epidemiology, variants of concern, digital polymerase chain reaction, Belgium, SARS-CoV-2

## Abstract

Since the beginning of the COVID-19 pandemic, the wastewater-based epidemiology (WBE) of SARS-CoV-2 has been used as a complementary indicator to follow up on the trends in the COVID-19 spread in Belgium and in many other countries. To further develop the use of WBE, a multiplex digital polymerase chain reaction (dPCR) assay was optimized, validated and applied for the measurement of emerging SARS-CoV-2 variants of concern (VOC) in influent wastewater (IWW) samples. Key mutations were targeted in the different VOC strains, including SΔ69/70 deletion, N501Y, SΔ241 and SΔ157. The presented bioanalytical method was able to distinguish between SARS-CoV-2 RNA originating from the wild-type and B.1.1.7, B.1.351 and B.1.617.2 variants. The dPCR assay proved to be sensitive enough to detect low concentrations of SARS-CoV-2 RNA in IWW since the limit of detection of the different targets ranged between 0.3 and 2.9 copies/µL. This developed WBE approach was applied to IWW samples originating from different Belgian locations and was able to monitor spatio-temporal changes in the presence of targeted VOC strains in the investigated communities. The present dPCR assay developments were realized to bring added-value to the current national WBE of COVID-19 by also having the spatio-temporal proportions of the VoC in presence in the wastewaters.

## 1. Introduction

Since the start of the COVID-19 pandemic, diverse genetic lineages of SARS-CoV-2 have emerged and circulated globally. Among these different lineages, some variants of concern (VOC) show higher transmissibility, higher disease severity, reduced susceptibility to treatment or vaccination, diagnostic detection failure or reduced neutralization by antibodies [1,2,3]. On 11 May 2021, the World Health Organization (WHO) designated four different VOC, including the B.1.1.7 (alpha lineage; first discovered in United Kingdom in September 2020), the B.1.351 (beta lineage; first occurred in South Africa in May 2020), the P.1 (gamma lineage; first described in Brazil in November 2020) and the B.1.617.2 (delta lineage; first discovered in India in October 2020) lineages [1]. Each VOC is characterized by a set of co-occurring mutations (Table 1) of which some could also potentially increase the virulence of SARS-CoV-2 [4,5].

Current detection methods for SARS-CoV-2 variants in clinical samples (e.g., nasal swabs) include quantitative reverse-transcription polymerase chain reaction (RT-qPCR) and for some selected samples of interest (i.e., mostly of symptomatic patients), next generation sequencing (NGS). Using NGS approaches, the entire genome of the variant can be sequenced, providing detailed information on the genotype. However, this is time consuming and expensive [7,8]. Additionally, RT-qPCR-based molecular assays have proved useful for the rapid detection of VOC [7]. Multiple methods have been developed targeting single-nucleotide polymorphisms (e.g., N501Y and E484K) or characteristic deletions (e.g., spike SΔ69/70 deletion and ORF1a Δ3675–3677) in the genome (often the spike domain) of the SARS-CoV-2 virus [7,8,9]. RT-qPCR strategies for SARS-CoV-2 detection focus often on indirect detection (for example, through drop-out signals) and could potentially result in the inclusion of false negative results. Additionally, most of these assays were designed to focus on a single VOC specifically. Furthermore, RT-qPCR results can be influenced by inhibition (i.e., by matrix interferences) and this only provides a relative quantification (i.e., through the use of a standard curve), which makes it difficult to compare results between laboratories and assays. In addition, the RT-qPCR makes it difficult to identify the relative occurrence of different VOC. The aforementioned limitations address the need for a complementary epidemiological approach, which ideally is based on a high-throughput, cost-effective molecular assay, capable of providing quantitative data. Additionally, the emergence of novel VOC highlights the need for continued surveillance to control the spread of SARS-CoV-2.

Since the feces and, to a lesser extent, urine of COVID-19 patients can contain fragments of the SARS-CoV-2 genome, viral RNA of infected people enters the wastewater system through human excretion [10,11,12]. Therefore, influent wastewater (IWW) samples can be analyzed for traces of SARS-CoV-2 RNA to monitor the resurgence and spread of SARS-CoV-2 infections at high spatial and temporal resolutions [13,14]. This type of surveillance system provides the possibility to monitor a large population with only one sample per sewer catchment area (i.e., delimited area covered by a unique sample of IWW). Since not only symptomatic, but also asymptomatic patients can be shedding SARS-CoV-2 RNA in their stool (and urine), wastewater-based epidemiology (WBE) can measure the true extent of (asymptomatic) infections and, therefore, include cases that might not be captured during clinical testing [13,15]. In addition, WBE can be considered more cost-efficient than clinical testing to monitor large populations. While clinical surveillance has the important advantage that it allows the identification and isolation of positive cases, it is limited to monitoring waves when patient case reporting is not uniform and when countries face restricted resources for clinical diagnosis and/or limited access to health care [16,17,18]. Additionally, WBE is a useful monitoring tool when clinical diagnoses are not constant in time, e.g., increase in testing during holidays, or during respiratory disease circulation. Therefore, WBE has proved to be a promising complementary tool for the surveillance and/or early warning system of disease outbreaks [13,19,20]. It also completes the global view of the sanitary situation and the resulting assessment communicated to decision makers. The applicability of WBE to monitor the circulation of SARS-CoV-2 was also demonstrated by the numerous population- and subcatchment-level monitoring studies during the past year [14,15,21,22,23,24].

WBE could potentially also be applied to provide a snapshot of the occurrence of different genetic lineages and their diversity at the population level [25,26,27,28,29]. However, the detection of different VOC in IWW samples presents also some challenges, including low concentrations of the RNA and the presence of PCR-inhibiting compounds in IWW [30,31]. Additionally, SARS-CoV-2 RNA fragments retrieved in IWW could originate from different viral lineages that are mixed into a single IWW sample representative for all variants circulating in the catchment area. Furthermore, the fecal shedding rate could be a variable for the different variants circulating in the general population [32,33]. For this reason, surveillance of the different SARS-CoV-2 VOC in IWW is complicated by tracking combinations of multiple characteristics and often co-occurring amino acid mutations [29].

The aim of this study was to optimize and validate a targeted multiplex digital PCR (dPCR) assay capable of detecting different VOC (B.1.1.7, B.1.351, P.1 and B.1.617.2) and furthermore providing an absolute quantification the RNA from different SARS-CoV-2 VOC in IWW. This bioanalytical approach was applied to IWW samples from different locations in Belgium to retrospectively monitor the emergence of each VOC at a population scale. To our knowledge, most pre-existing WBE applications on SARS-CoV-2 variants mainly focus on sequencing [29,34,35,36,37] and RT-qPCR [26,28,38,39]. Only a limited number of studies have applied dPCR to estimate the VOC circulation in IWW [25,27,40,41]. In addition, most pre-existing dPCR assays for WBE only focus on a limited number of targets [25,39,40]. For this reason, our aim was to include variant specific primers and probes capable of discriminating between the different VOC lineages present in a single IWW sample.

## 2. Materials and Methods

### 2.1. Sampling

Daily 24 h composite IWW samples have been collected twice per week (i.e., Monday and Wednesday) starting from September 2020 in context of the ongoing national wastewater surveillance program coordinated by the Belgian Scientific Institute for Public Health (Sciensano). A total of 42 locations covering around 45% of Belgian population are being monitored for this national wastewater surveillance of COVID-19 (public dashboard: https://datastudio.google.com/embed/u/0/reporting/c14a5cfc-cab7-4812-848c-0369173148ab/page/p_ggbfgsqtmc; accessed on 7 February 2022). A minor selection of samples that tested positive for the N1, N2 and E gene of SARS-CoV-2 were selected for the optimization and validation of the dPCR assay (collected between October 2020 and November 2021; thus, before the occurrence of the Omicron variant). This selection was made based on the timeframe in which a specific VOC was circulating and on the positivity rate in the different locations. Population equivalents in the selected locations ranged between 25,000 and 200,000 inhabitants. Important to note is that the IWW matrix composition differs substantially between locations of interest to demonstrate the robustness of the dPCR assay. Although it is virtually impossible to measure the wide range of matrix effects present in IWW, the matrix composition will differ significantly across time and locations due to the spatio-temporal differences in the disposal and excretion of compounds in IWW [42]. Locations are not further specified due to anonymity constraints.

The average travel time during in-sewer transport was less than 12 h in all locations and the pH of the samples remained neutral throughout the entire sampling period. Samples were taken either flow- or time-proportionally. In the case of the time-proportional sample collection, a high frequency (less than 10 min) was used to compose the daily IWW samples. Then, 500 mL wastewater was immediately stored after collection at 4 °C to prevent in-sample degradation of SARS-CoV-2 RNA [43,44]. At 24 h upon sample collection, RNA extraction of the IWW samples was performed. Generated extracts were stored at -80 °C until analysis (see Section 2.3).

### 2.2. In Silico Screening dPCR Primer Sets

SnapGene (GSL Biotech LLC, Chicago, IL, USA) was used for the preliminary in silico screening of primers and probes found in the literature [7,26,28,45]. The binding of each primer and probe on the genomic sequences of the different SARS-CoV-2 lineages was screened with this software. In particular, the specificity of each primer set against the respective VOC was tested. An overview of the final primer and probes list can be found in Table 2. All primers and probes were purchased from Integrated DNA Technologies (IDT, Leuven, Belgium). The original sequence of SARS-CoV-2 (MN908947.3) was retrieved from the NCBI database and the sequences for the B.1.1.7 (EPI_ISL_744131), the B.1.351 (EPI_ISL_660190), the P.1 (EPI_ISL_1121316) and B.1.617.2 (EPI_ISL_1704637) genome were taken from the GISAID database [4].

After this preliminary screening, the in silico inclusivity and false negatives of the assays used in this study were further evaluated using an in-house developed R script using R-software (RStudio 1.0.153; R3.6.1) and recent whole-genome SARS-CoV-2 sequences. SARS-CoV-2 genomes from samples from different species collected between 24 December 2019 and 30 December 2021 were obtained from the GISAID database on 2 January 2022 [47]. It should be noted that the database also includes sequences from other species than humans, such as bats, tigers, dogs and other animals, and a bat genome was included from 24 July 2013. Genomes containing undetermined nucleotides “N” and degenerate nucleotides were excluded from the dataset to retain only high-quality genomes. Moreover, genomes with a length below 29 300 nucleotides were excluded from the dataset, resulting in the inclusion of 2,391,563 SARS-CoV-2 genomes. From this dataset, the primers and probes were matched against the sequences (see Table S1). Sequences that had at least one mismatch were classified as a false negative result. Inclusivity and false negative results were obtained for all sequences and for each variant.

### 2.3. Sample Preparation and RNA Extraction

The extraction of SARS-CoV-2 RNA was performed according to a previously validated bioanalytical method [43]. Briefly, 20 mL of IWW was centrifuged at 4000× *g* for 30 min to remove solid particles. Subsequently, the supernatant was loaded on a Macrosep Advance Centrifugal device with Omega Membrane (100 kDa) and centrifuged for 30 min at 4600× *g*. Finally, the concentrate was extracted from the centrifugal filter and diluted to 1000 µL with ultrapure DNase/RNase free distilled water. RNA extraction was performed with the automated Maxwell PureFood GMO and Authentication RNA extraction kit. The final elution volume with this RNA extraction kit was 65 µL.

### 2.4. Molecular Assays: RT-qPCR and dPCR

RT-qPCR was performed during method optimization to compare its performance with dPCR. RT-qPCR was executed with the Lightcycler 480 instrument from Roche (Bazel, Switzerland). All RT-qPCR amplifications were done in 20 µL reaction mixtures with a SensiFAST^TM^ Probe No-ROX One-Step kit from Bioline (Cincinnati, OH, USA). The concentration of RNA within each reaction mixture was 20% *v*/*v* (4 µL template). All RT-qPCR reactions were performed in duplicate and settings were as follows: 10 min for reverse transcription at 45 °C, 2 min at 95 °C for polymerase activation followed by 45 cycles of 5 s at 95 °C for denaturation and 30 s at 60 °C for annealing and extension.

dPCR carried out with a QIAcuity Digital PCR System from QIAGEN (Hilden, Belgium) was used for absolute quantification of SARS-CoV-2 RNA concentrations. RNA extracts were assayed with a QiAcuity One-Step Viral RT-PCR kit (QIAGEN, Hilden, Belgium). Then, 10 µL of viral RNA was diluted to 40 µL with dPCR mastermix. dPCR settings were as follows: 40 min for reverse transcription at 50 °C, 2 min at 95 °C for polymerase activation followed by 40 cycles of 5 s at 95 °C for denaturation and 30 s at 60 °C for annealing and extension. A negative dPCR control was also amplified with dPCR to set the fluorescence threshold for each primer set. An overview of all dPCR targets and final primers and probes concentrations is given in Table 2.

### 2.5. Validation of the Specificity of the PCR Assays for the Different VOC Strains

**Figure 1:**
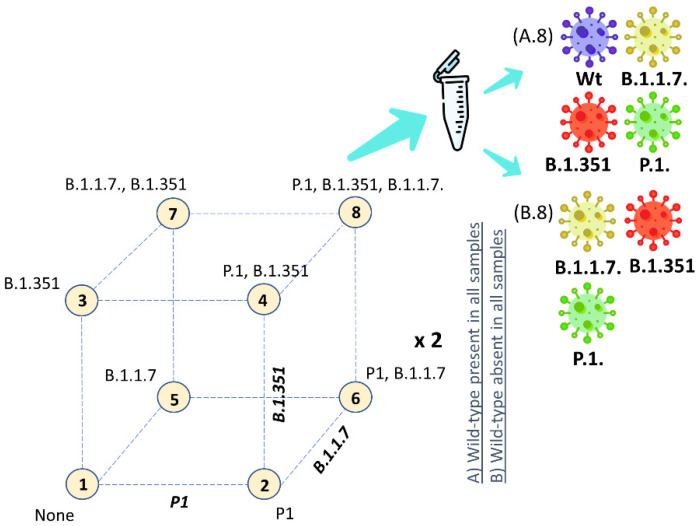
Design of experiment (DOE) for the preparation of the reaction mixtures containing different combinations of RNA of the variants of concern. Each corner of the cube represents a specific reaction mixture. The same DOE was applied with either (**A**) the Wuhan strain present in all samples or (**B**) the Wuhan strain absent in all samples.

The SARS-CoV-2 RNA of the Wuhan strain and the different VOC lineages used as a positive PCR control was obtained from the Institute of Tropical Medicine Antwerp (kind gift of Prof. K. Ariën, ITG, Belgium). To confirm the suitability of the PCR methods to distinguish and estimate the proportion of the wild-type and the different VOC targets, an experimental design (DOE) was executed with reaction mixtures containing different combinations of the SARS-CoV-2 VOC (as illustrated by Figure 1). For the P.1 and B.1.617.2 primer set, a slightly different RT-qPCR set-up was applied since primer sets and/or PCR controls were only available at a later time. In this set-up, each RT-qPCR assay was tested on the different RT-qPCR controls of each VOC to assess the specificity. Each sample only contained one RT-qPCR control. With dPCR, the B.1.617.2 primer set was initially tested on the B.1.617.2 RNA control to test if the primer set was capable of assaying its respective target. In the next step, the same primer set was tested on the RNA controls of the remaining VOC lineage applying the DOE as presented in Figure 1. This DOE was also applied to the P.1 primer set with dPCR.

In first instance, RT-qPCR was applied to optimize the PCR amplification for the available primer sets. A 10-fold serial dilution of the RNA of each SARS-CoV-2 strain was run to investigate RT-qPCR efficiency for the different primers. The results of this experiment proved to be acceptable, and a reproducible increase in Ct-values was observed when further diluting the positive control. This experiment was also performed with different annealing temperatures (ranging from 56 to 60 °C) to determine the optimal PCR settings. The final annealing temperature was set at 60 °C. Additionally, the concentrations of the primers and probes were also optimized. The final concentrations of the primers and probes can be found in Table 2. Subsequently, the different reaction mixtures of the SARS-CoV-2 lineages were assayed with these optimal settings (see Section 2.4), using RT-qPCR to investigate the specificity of the different primer sets. The same experimental set-up was processed with dPCR to compare the selectivity and sensitivity of both molecular assays.

### 2.6. Validation of Sensitivity of the dPCR Assay for the Different VOC Strains

The determination of the sensitivity of the selected VOC specific primer sets was carried out using serial dilutions of the corresponding positive PCR controls, as previously described by Van Poelvoorde et al. [48]. Seven serial dilutions were prepared for each primer set with concentrations ranging between 0.1 and 400 copies/µL, with minor differences for each primer set based on the starting concentration of the positive RNA control. Each serial dilution was prepared in triplicate. For each primer set, the limit of detection (LOD95%) was calculated with the webtool Quodata with the copy number of each target that yields in a probability of detection (POD) of 95% [48,49].

## 3. Results and Discussion

### 3.1. Identification and In Silico Inclusivity Evaluation of Specific PCR Primer Sets

The final selection of PCR primers and probes can be found in Table 2. The primer sets given in this table proved to be specific for their corresponding SARS-CoV-2 strain after in silico screening. The described targeted dPCR assay focuses on characteristic mutations within the SARS-CoV-2 genome. This is necessary since mutant strains are almost completely identical to the wild-type with only minor single-nucleotide polymorphisms, deletions and/or insertions. For example, the spike region of the B.1.1.7 variant and the original sequence are almost identical, apart from six nucleotide deletions. For this reason, both the SΔ69/70 deletion specific and B.1.1.7 specific primer sets were focused on the SΔ69/70 deletion of the spike region for the detection and discrimination of the B.1.1.7 variant, respectively [7,26]. Additionally, the probe of the B.1.351 strain was located on the Δ241 in the spike region characterizing this specific variant. Therefore, this primer set should be able to distinguish the B.1.351 lineage from other VOC and wild types [26]. The probe of the B.1.617.2 variant was designed to assay a characteristic deletion 157–158 in the spike region of SARS-CoV-2, which is absent in other VOC lineages [28]. In contrast to the primer sets for the other VOC in this study, the probe design of the P.1 variant did not focus on the spike domain of the SARS-CoV-2 genome but targeted the end of the ORF8 and beginning of the N gene. Within this region the P.1 variant is almost completely the same as the Wuhan-1 strain, with the exception of a 4-nucleotide insertion [28]. Finally, the N501Y primer set targets the N501Y mutation in the spike region of the SARS-CoV-2 genome, which is present in the B.1.1.7, the B.1.351 and P.1 strain [6,45].

The inclusivity of all assays was evaluated for all sequences and for each targeted variant defined by GISAID. A detailed overview of the results of this in-silico screening is given in Tables S1 and S2.

The SΔ69/70 deletion assay targets the lack of signal at the SΔ69/70 deletion. This deletion is characteristic for genomes of the B.1.1.7 variant. For both primers, an inclusivity of more than 99.0% was observed for sequences defined as the B.1.1.7 variant. Moreover, in 99.9% of the B.1.1.7 genomes, no match was observed with the probe of this assay, which would result in a signal drop-out. In 58 other variants, this signal drop-out would also be observed in at least 50.0% of the genomes of that variant. These variants include the variant of concern, B.1.1.529, and variants of interest, B.1.620 and B.1.525.

The B.1.1.7 assay targets the SΔ69/70 deletion, which is characteristic for genomes of the B.1.1.7 variant. Both primers have an inclusivity of more than 99.0%, while for the probe, an inclusivity of 98.9% was observed within sequences attributed to the B.1.1.7 variant. Furthermore, this probe matches with 35 other variants, of which at least 50.0% of the genomes of that variant match with the probe. These variants include the variant of interest B.1.620.

The N501Y assay is characteristic for genomic variants containing this mutation. The target mutation is situated within the forward primer, and an inclusivity of 99.9% was observed for sequences defined as the B.1.1.7 variant. Moreover, an inclusivity of more than 99.5% was observed for the reverse primer and probe. However, the forward primer also matches with 48 other variants of which at least 50% of the genomes of that variant match with the forward primer. These variants include variants of concern B.1.351, P.1 and variant of interest B.1.621.

The B.1.351 assay targets the SΔ241 deletion, which is characteristic for genomes of the B.1.351 variant. An inclusivity of more than 98.0% was observed for both primers, while an inclusivity of 96.2% was observed with the probe within sequences defined as the B.1.351 variant. The other variants of which at least 50.0% of the genomes match with the probe are B.1.351.2, B.1.351.3, B.1.351.5, which are sublineages of the B.1.351 variant.

The B.1.617.2 assay targets the SΔ157 deletion, which is characteristic for genomes attributed to the B.1.617.2 variant. An inclusivity of more than 99.0% was observed for both primers, while an inclusivity of 97.7% was observed with the probe within sequences defined as the B.1.617.2 variant. The probe also matches with 183 other variants of which at least 50.0% of the genomes of that variant match with the probe. These variants mostly include sublineages of B.1.617.2 and B.1.617.3.

### 3.2. Validation of the Specificity of the PCR Primer Sets

As highlighted in Section 3.1, it is not straightforward to identify specific target sites in the genome of the different VOC strains to assay these mutants specifically since the respective mutations could potentially be shared by other existing lineages. However, it should be noted that the circulation of other (unknown) variants is expected to be much lower compared to the VOC strains. This was also confirmed by genomic surveillance of SARS-CoV-2 in Belgium with a combined detection frequency of less than 5% for other variant strains [50]. Since the concentration of SARS-CoV-2 RNA in IWW is generally low (generally 1–100 copies/µL range), it is expected that these other strains will have a less prominent share compared to the circulating VOC strains. For this reason, the main focus was to validate the specificity of the different primer sets on the RNA of the VOC lineages that were prominent between October 2020 and November 2021.

#### 3.2.1. RT-qPCR

**Figure 2:**
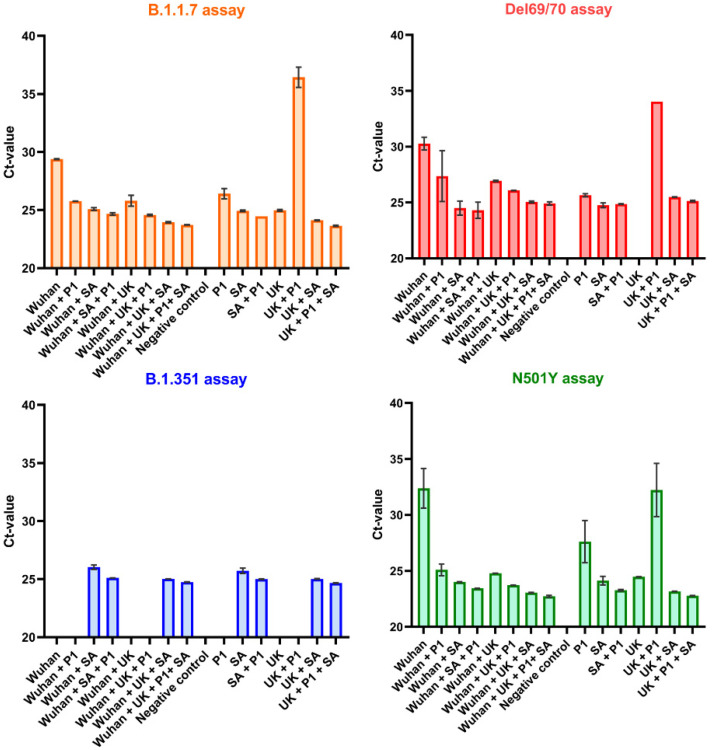
Specificity of the different primer sets (B.1.1.7 = orange; SΔ69/70 = red; B.1.351 = blue; and N501Y assay = green) to reaction mixtures (*x*-axis) containing different combinations of variants of concern RNA with real-time quantitative PCR. The experiment was performed in duplicate and the mean Ct-value was plotted for each reaction mixture. (Wuhan = wild type; UK = B.1.1.7; SA = B.1.351).

In the first instance, the different VOC combinations (as discussed in Section 2.5.) were assayed with RT-qPCR to test the specificity of the primer sets to their respective SARS-CoV-2 lineages. As illustrated in Figure 2, the primer set designed for the B.1.351 variant proved to be selective with RT-qPCR. A positive signal was only observed in the reaction mixtures containing the B.1351 genome. Additionally, Ct-values were reproducible across the different reaction wells containing the same concentration of RNA. The primer set targeting the spike SΔ69/70 deletion resulted in a negative response (spike “drop-out”) in the reaction mixture that only contained the B.1.1.7 lineage but resulted in positive Ct-values in the reaction wells containing the wild-type and other VOC lineages. For this reason, this spike “drop-out” signal can be used to obtain information on the occurrence of the B.1.1.7 VOC in biological samples if used in combination with the other primer sets.

The primer sets targeting the B.1.1.7 VOC specifically did not prove to be selective during this experimental set-up with RT-qPCR. However, a higher intensity of the fluorescence signal was observed with RT-qPCR for the amplification curves of the reaction wells containing the B.1.1.7 genome. Only a weak fluorescence signal (i.e., 5 times lower) was observed in the reaction mixtures without the B1.1.7 lineage. This indicates that further optimization of the B1.1.7 primer sets with dPCR could potentially result in higher selectivity.

As discussed earlier, the N501Y primer set should yield in a positive signal for the different VOC lineages containing the N501Y mutation (i.e., B.1.1.7, B.1.351 and P.1), while this primer set should be negative for the Wuhan strain and B.1.617.2 strain. For this reason, these primer sets should be capable of detecting the presence of combinations of the B1.1.7, B1.351 and P.1 variants in biological samples. As illustrated in Figure 2, the use of the N501Y primer sets also resulted in a positive signal in the reaction well containing the Wuhan strain. However, the intensity of the amplification curves measured in the reaction mixtures containing the VOC lineages was higher compared to fluorescent signal observed in the well containing the wild type. Further optimization of the N501Y primer sets was, therefore, required.

In contrast to the B. 1.1.7 and N501Y primer set, the assays for P1 and B.1.617.2 VOC were found specific with the RT-qPCR method. In both cases, there was only a fluorescent signal observed for the corresponding VOC. It should be noted that a slightly different set up was used for these primer sets, as discussed in Section 2.5.

#### 3.2.2. dPCR

The same experimental design was conducted with dPCR to further investigate the applicability of the N501Y, B.1.1.7, P.1 and B.1.617.2 primer sets. The sensitivity of dPCR could potentially be higher compared to RT-qPCR due to the higher tolerance for PCR inhibitors [51,52,53]. In IWW with low RNA concentrations, RT-qPCR could potentially be influenced by a wide array of organic matter and heavy metals present in the biological matrix, resulting in poor amplification efficiency, lower precision and need for a standard curve for relative quantification [53,54,55]. With dPCR, the sample is partitioned in a high number (approximately 26,000) of separate self-contained reaction wells. An end-point dPCR assay is performed in each of these reaction chambers. The partitioning of SARS-CoV-2 RNA in the different reaction wells makes it possible to simultaneously discriminate and enumerate the low frequencies of VOC and wild-type fragments [25,40]. This could potentially result in a higher specificity for variant detection, compared to RT-qPCR.

As discussed before, with dPCR, the sample and master mixes are partitioned in a large number of reaction chambers (~26,000) and the viral RNA in biological samples is assayed in separate reactions. This could potentially result in higher specificity with dPCR, compared to RT-qPCR. In contrast to the RT-qPCR assay, the B.1.1.7 primer set proved to be specific for the B.1.1.7 variant with dPCR further confirming the higher dPCR specificity. Combinations that did not contain the B.1.1.7 variant were negative during the dPCR assay, while a low fluorescent signal was observed with RT-qPCR (see Section 3.2.1). Concentrations of the B1.1.7 RNA were similar in the different reaction mixtures further indicating that other VOC lineages were not assayed in the wells containing a multiple number of variants.

**Figure 3:**
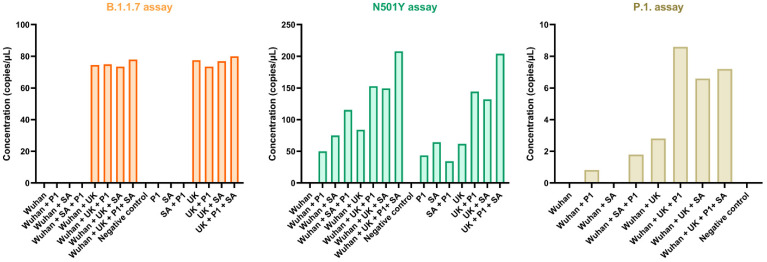
Specificity of the B.1.1.7 (orange), the N501Y (green) and P.1. (brown) assay to reaction mixtures (*x*-axis) containing different combinations of variants of concern RNA with digital PCR.

In addition, the N501Y primer set was selective for the VOC lineages containing the N501Y mutation and did not result in a positive signal for the Wuhan strain (see Figure 3). For this reason, the dPCR assay is capable of measuring a combined signal of all VOC lineages containing the N501Y mutation through the use of this primer set. The B.1351 and the SΔ69/70 deletion primer sets also proved to be specific with dPCR.

The B.1.617.2 primer set proved to be capable of assaying the RNA of the B.1.617.2 strain. Additionally, the use of B.1.617.2 primer set with the reaction mixtures of the DOE did not yield in any positive results, which indicates that there is no interference with the other VOC strains. The use of the B.1.1.7, N501Y and B.1.351 primer sets did not give a positive test result for the B.1.617.2, further indicating the specificity of the different primer sets for the different VOC lineages. As expected, the SΔ69/70 deletion primer set tested positive for the B.1.617.2 strain. These results highlight the specificity of the B.1.617.2 primer set in assaying the RNA of the corresponding VOC.

As illustrated by Figure 3, the P.1 primer set tested negative on the RNA of the Wuhan and B.1.351 strain but tested positive for both the B.1.1.7 and P.1 strain. This indicates that this primer set was not specific to assay P.1 in particular. For this reason, this primer set was excluded from the assay, and the occurrence of the P.1 VOC was measured through the application of the N501Y assay specifically. No further selectivity assessment and application to IWW was done with the P.1 primer set, due to interference with the B.1.1.7 RNA.

In conclusion, the final assay is able to distinguish between the wild-type, B.1.1.7, B.1351, B.1.617.2 and other lineages containing the N501Y mutation (e.g., P.1) in the spike genome of SARS-CoV-2. The inclusion of a spike “drop-out” signal resulting in target failure for the B.1.1.7 VOC is also possible through the use of the SΔ69/70 deletion primer set. The E-gene tested positive for the wild-type and the different VOC.

### 3.3. Validation of the Sensitivity of the dPCR Assay

The sensitivity of the P.1 primer set was not further tested, as it was not specific for its corresponding VOC lineage with the RT-qPCR method. It should be noted that the sensitivity of the N501Y assay was only tested on the RNA control of B.1.1.7 lineage and not on the B.1.351 and P.1 strains. This was done since the B.1.1.7 was the most dominant VOC prior to the emergence of the B.1.617.2 lineage. Additionally, the N501Y primer set did not target the B.1.617.2. strain. The SΔ69/70 deletion primer set yields a negative “drop-out” signal for the B.1.1.7 strain, but in a positive signal for the remaining VOC. For this reason, the sensitivity of this assay was tested on the B.1.617.2. lineage since it was the most dominant VOC in the Belgian population at the time of the analysis.

The sensitivity of the dPCR assay was validated by using the RNA of the different VOC strains with different estimated target copy numbers, ranging between 0.1 and 400 copies/µL for all VOC. The results of this evaluation can be found in Table 3. The LOD95% was below 3 copies/µL for all dPCR targets, which is acceptable since SARS-CoV-2 levels in IWW are generally in the low copies/µL range [14,26,43], as reflected by the data in Section 3.4. Overall, the different primer sets showed good reproducibility at the low concentration levels of the serial dilution, even with only three replicates included. However, it should be noted that the variation at detection limits was higher for the B.1.617.2 primer set compared to the other dPCR targets, which explains the broader range of the 95% confidence interval for the LOD95%. This could potentially also be explained by the larger concentration difference between concentration level 6 and level 7 compared to the serial dilution of the LOD95% assessment for the other targets (Table 3 and Table S3).

### 3.4. Detection of the Different SARS-CoV-2 Variants of Concern in Belgian Influent Wastewaters Using the Validated dPCR Assay

**Figure 4:**
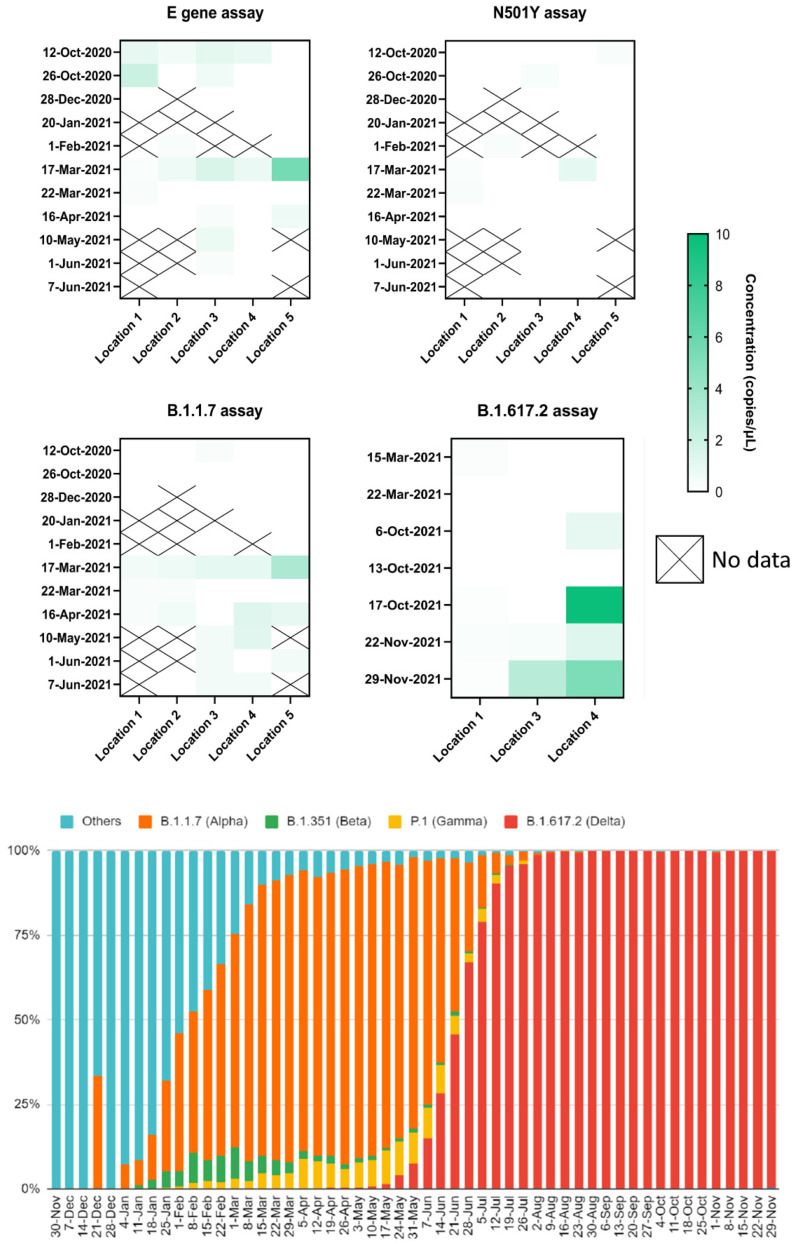
Application of the digital PCR assay to influent wastewater samples originating from 5 Belgian wastewater treatment plants. Information on the genomic surveillance of SARS-CoV-2 was adopted from [50]. No sample was analyzed for the dates with ‘X’ since these timepoints were negative during the national wastewater surveillance program. For the same reason, locations 2 and 5 were not included for the evaluation of the B.1.617.2 assay.

IWW samples were selected between 12 October 2020 and 29 November 2021 to investigate the presence of VOC in different Belgian catchment areas. Figure 4 shows temporal patterns in the occurrence of the wild-type and VOC lineages in Belgium based on wastewater data (top half). In parallel, this figure displays the results of the genomic surveillance of SARS-CoV-2 (bottom half) [50]. In this study, the E gene assay was used as an indicator for the presence of SARS-CoV-2 RNA in IWW. Its applicability was proven before in the Belgian national wastewater surveillance program. In this study, the different IWW samples tested positive throughout the entire time horizon of the sampling campaign for the E gene. With this E assay alone, it is not possible to distinguish between the different VOC lineages and the addition of information from the variant-specific dPCR assays is necessary for the identification of the most important VOC strains circulating in the different Belgian communities.

The N501Y assay targets all VOC strains that contain this respective mutation, for example, B.1.1.7, B.1.351 and P.1. In theory, this assay should be able to monitor the transition from wild type to B.1.1.7 and should be negative at the time of B.1.617.2 circulation. Indeed, the positivity rate was higher with this assay at the beginning of March after an increase in B.1.1.7 cases was observed. It was negative at the beginning of June when B.1.617.2 cases emerged. However, it should be noted that the N501Y assay was negative in most cases. Overall, native concentrations found with the N501Y primer set were also lower compared to the primer sets targeting the E gene, the B1.1.7 and the B.1.617.2 lineage. We hypothesize that this primer set is less sensitive in IWW compared to the others. The optimization of the PCR amplification was done for each primer set individually. Although annealing temperatures were optimized for all primers separately, optimal dPCR settings were the same for all primer sets. Concentrations of the primer sets in the master mixes for dPCR were also optimized for each of the primer sets. The imaging step was finetuned for each of the fluorophores. However, the LOD95% in DEPC treated water was in line with the other dPCR targets that were measured in this timeframe. The lower sensitivity of N501Y in IWW could potentially also be linked to the complexity of the wastewater matrix. IWW samples contain a broad range of matrix interferences that could potentially interfere with the dPCR assay. The N501Y primer sets could potentially be influenced to a larger extent, compared to the other PCR primers. Furthermore, substantial variability at these lower limit of quantification (LLOQ) levels should also be taken into account, as previously indicated by others [14,43,44].

The steep increase in detection levels of B.1.1.7 RNA in March 2021 confirms the applicability of this dPCR in detecting and quantifying this VOC in IWW. Additionally, starting from 17 March 2021, RNA concentrations of the E gene were in line with the B1.1.7 lineage, further evidencing that this variant was the most dominant strain of SARS-CoV-2 circulating at that moment. This is the case since the primer set targeting the envelope yields a positive signal for both the wild-type and the VOC lineages. RNA concentrations for the E gene in late 2020 most likely originate from the presence of the wild type in IWW since the majority of samples were negative for the B1.1.7, the B1.351 and the N501Y primer set. This pattern is also reflected by the genomic surveillance data provided by the National Reference Centre.

It should be noted that the B.1.351 lineage (Beta) was not found in any of the IWW samples, which could be explained due to the fact that the circulation of this VOC in Belgium was lower compared to the other VOC lineages. Indeed, genomic sequencing of SARS-CoV-2 showed that the circulation of the B.1.351 strain was lower in Belgium compared to the other SARS-CoV-2 lineages (<10%). In the IWW sample of 17 March 2021 and later on, there was an increase in the RNA concentration of the B.1.1.7 lineage. This variant was not detected in any of the previous IWW samples with the exception of 12 October 2020 in location 3. However, this result originated from a very low signal and could therefore potentially be false positive. Although the B.1.1.7 mutant was found in clinical samples in September 2020 in the United Kingdom, this lineage only received its status as VOC in December 2020 and genomic surveillance of SARS-CoV-2 reported the occurrence of the B.1.1.7 strain for the first time on 21 December 2020, being further suggestive of a false positive signal in our assays.

Since the B.1.617.2 primer set was only available at a different time, a similar experiment was conducted at a later instance focusing on the dates before and after the resurgence of this VOC strain. As shown by Figure 4, the B.1.617.2 tested predominantly positive in October 2021 and November 2021 when B.1.617.2 was the most dominant strain. No positive test results were observed in the IWW samples of March 2020, which would be expected since the B.1.617.2 strain was reported for the first time in April 2020 in Belgium. In the same experiment, the samples from locations 1 and 3 and locations 1 and 4 tested positive for the B.1.1.7 strain on 15 March 2021 and 22 March 2021, respectively. No positive test results were found for the B.1.1.7 assay in the IWW samples from October 2021 and November 2021, further highlighting the shift from the B.1.1.7 strain to the B.1.617.2 strain. It should be noted that the B.1.1.7. could potentially yield positive test results for the B.1.1.529 strain. However, this SARS-CoV-2 VOC was not detected in Belgium during the timeframe of this study. In the same experiment, the B.1.351 assay yielded only negative test results.

Measured concentrations found in this study were in line with the results found in other studies [26,28,38]. This emphasizes the applicability of the presented bioanalytical method to measure low concentrations of SARS-CoV-2 RNA present in IWW samples.

## 4. Conclusions

The present study presents a complementary epidemiological method, which is wastewater- and dPCR-based, for the surveillance of different VOC in the general population. The proposed WBE approach could be applied to provide a snapshot of the presence of different SARS-CoV-2 strains and their diversity at the population scale. The targeted multiplex dPCR assay proved to be specific for the B.1.1.7, the B.1.351 and the B.1.617.2 strains in IWW. Primer sets targeting the N501Y were also included to monitor the occurrence of remaining lineages containing this mutation (e.g., P.1, B.1.1.7, and B.1.351). The LOD95% of the different dPCR targets ranged between 0.3 and 2.9 copies/µL, proving its applicability to measure the low detection levels of SARS-CoV-2 RNA present in IWW. Additionally, the proposed bioanalytical method proved capable of monitoring spatio-temporal changes in the presence of different VOC lineages through the analysis of IWW in different Belgian communities. Furthermore, this assay could be employed complementarily to the national surveillance of SARS-CoV-2 in Belgium and could also be adopted in other European countries for the detection of different SARS-CoV-2 variants in IWW. A similar development and validation could also be employed for the omicron variant.

## Figures and Tables

**Table 1:** Overview of the variants of concern and key mutations. (*): Source of date of earliest detection: https://www.who.int/en/activities/tracking-SARS-CoV-2-variants/ (accessed on 7 February 2022).

WHO Label	Pango Lineage	GISAID Clade	Key Mutations in Spike Gene [6]	Earliest Detection in Samples *
Alpha	B.1.1.7	GRY	N501Y D614G SΔ69/70 P681H Y144 del A570D T716I S982A D1118H	United Kingdom, September 2020
Beta	B.1.351	GH/501Y.V2	N501Y E484K K417N	South Africa, May 2020
Gamma	P.1	GR/501Y.V3	N501Y E484K K417N D614G	Brazil, November 2020
Delta	B.1.617.2	G/478K.V1	P681R L452R T478K T19R Del156-157 R158G D614G D95N	India, October 2020

**Table 2:** List of dPCR targets.

Target Gene Fragment	Primer/Probe	Final Concentration (Nm)	5′	Sequence	3′	Targeted Mutations	Amplicon Size (Bp)	Ref
Enveloppe (E)	E_Sarbeco_F	400	None	ACAGGTACGTTAATAGTTAATAGCGT	None	Not Applicable	113	[46]
	E_Sarbeco_R	400	None	ATATTGCAGCAGTACGCACACA	None			
	E_Sarbeco_P1	200	/FAM/	ACACTAGCCATCCTTACTGCGCTTCG	/ZEN//3IaBkFQ/			
Spike (S)	Del69/70-Forward	200	None	TCAACTCAGGACTTGTTCTTACCT	None	SΔ69/70 (drop-out signal)	102	[7]
	Del69/70-Reverse	200	None	TGGTAGGACAGGGTTATCAAAC	None			
	Del69/70-probe	200	/5HEX/	TTCCATGCTATACATGTCTCTGGGA	/ZEN//3IaBkFQ/			
	N501YMutation-Forward	200	None	CATATGGTTTCCAACCCACTT	None	N501Y	82	[45]
	N501YMutation-Reverse	200	None	GGTGCATGTAGAAGTTCAAAAGAAAGT	None			
	N501YMutation-Probe	200	/5Cy5/	TGGTGTTGGTTACCAACCATACAGAG	/3IAbRQSp/			
	B1.351_Specific-Forward	200	None	AGATTTGCCAATAGGTATTAACATC	None	SΔ241	80	[26]
	B1.351_Specific-Reverse	200	None	CTGAAGAAGAATCACCAGGAGTC	None			
	B1.351_Specific-Probe	200	/5TexRd-XN/	CTAGGTTTCAAACTTTACATAGAAGTT	/3IAbRQSp/			
	B1.1.7_Specific-Forward	200	None	GTTCTTACCTTTCTTTTCCAATGTTAC	None	SΔ69/70	99	
	B1.1.7_Specific-Reverse	200	None	CCATCATTAAATGGTAGGACAGGG	None			
	B1.1.7_pecific-Probe	200	/5HEX/	TGGTTCCATGCTATCTCTGGGACC	/ZEN//3IaBkFQ/			
	Delta-F21989	200	None	GTTTATTACCACAAAAACAACAAAAG	None	SΔ157	95	[28]
	Delta-R22083	200	None	GGCTGAGAGACATATTCAAAAGTG	None			
	S157	200	/5Cy5/	TGGATGGAAAGTGGAGTTTATTCTAGT	/ZEN//3IaBkFQ/			
ORF8/Nucleocapside (N)	P.1_specific-Forward	200	None	CATGACGTTCGTGTTGTTTTAG	None	Insertion in 28227–28286 region	85	
	P.1_Specific-Reverse	200	None	CATTTCGCTGATTTTGGGGTCC	None			
	P.1.-P	200	/5HEX/	TTTCATCTA/ZEN/ AACGAACAAACAAACAAACTAAAAT	/ZEN//3IaBkFQ/			

**Table 3:** Assessment of the limit of detection (LOD95%) of the different variant specific primer sets.

	Del69/70	N501Y	B.1.1.7	B.1.351	B.1.617.2
LOD95% [95% confidence interval]	<0.5	0.3 [0.1; 1.7]	<0.4	0.4 [0.2; 1.3]	2.9 [0.4; 20.5]

## Data Availability

All data are available in the manuscript and Supplementary Materials.

## Supplementary Materials

The following are available online at https://www.mdpi.com/article/10.3390/v14030610/s1, Table S1: In-silico inclusivity and specificity of the different PCR targets; Table S2: In-silico specificity of the final dPCR target list on the different variants of concern (VOC) and variants of interest (VOI); Table S3: Assessment of the LOD95% of the different variant specific primer sets. For each concentration level, the positive detection rate (%) among replicates was given in italics together with the mean copy number (copies/µL ± standard deviation) as observed with the dPCR method.

## References

[1] World Health Organization (WHO) Tracking of SARS-CoV-2 Variants. https://www.who.int/en/activities/tracking-SARS-CoV-2-variants/.

[2] Centers for Disease Control and Prevention SARS-CoV-2 Variant Classifications and Definitions. https://www.cdc.gov/coronavirus/2019-ncov/variants/variant-info.html.

[3] Sanyaolu A., Okorie C., Marinkovic A., Haider N., Abbasi A.F., Jaferi U., Prakash S., Balendra V. (2021). The emerging SARS-CoV-2 variants of concern. Ther. Adv. Infect. Dis..

[4] GISAID Tracking of Variants. https://www.gisaid.org/hcov19-variants/.

[5] Hadfield J., Bedford T., Neher R., Hodcroft E., Sibley T., Huddleston J., Aksamentov I., Lee J., Fay K., Zuber M. Nextstrain: Real-Time Tracking of Pathogen Evolution. https://nextstrain.org/ncov/#sit-reps.

[6] Lopez-Rincon A., Perez-Romero C.A., Tonda A., Mendoza-Maldonado L., Claassen E., Garssen J., Kraneveld A.D. (2021). Design of Specific Primer Sets for the Detection of B.1.1.7, B.1.351, P.1, B.1.617.2 and B.1.1.519 Variants of SARS-CoV-2 Using Artificial Intelligence. bioRxiv.

[7] Vogels C.B.F., Breban M.I., Ott I.M., Alpert T., Petrone M.E., Watkins A.E., Kalinich C.C., Earnest R., Rothman J.E., Goes de Jesus J. (2021). Multiplex QPCR Discriminates Variants of Concern to Enhance Global Surveillance of SARS-CoV-2. PLoS Biol..

[8] Chaintoutis S.C., Chassalevris T., Tsiolas G., Balaska S., Vlatakis I., Mouchtaropoulou E., Siarkou V.I., Tychala A., Koutsioulis D., Skoura L. (2021). A One-Step Real-Time RT-PCR Assay for Simultaneous Typing of SARS-CoV-2 Mutations Associated with the E484K and N501Y Spike Protein Amino-Acid Substitutions. medRxiv.

[9] Bedotto M., Fournier P.-E., Houhamdi L., Levasseur A., Delerce J., Pinault L., Padane A., Chamieh A., Tissot-Dupont H., Brouqui P. (2021). Implementation of an in-house real-time reverse transcription-PCR assay for the rapid detection of the SARS-CoV-2 Marseille-4 variant. J. Clin. Virol..

[10] Chen Y., Chen L., Deng Q., Zhang G., Wu K., Ni L., Yang Y., Liu B., Wang W., Wei C. (2020). The presence of SARS-CoV-2 RNA in the feces of COVID-19 patients. J. Med. Virol..

[11] Lescure F.-X., Bouadma L., Nguyen D., Parisey M., Wicky P.-H., Behillil S., Gaymard A., Bouscambert-Duchamp M., Donati F., Le Hingrat Q. (2020). Clinical and virological data of the first cases of COVID-19 in Europe: A case series. Lancet Infect. Dis..

[12] Zhang J., Wang S., Xue Y. (2020). Fecal specimen diagnosis 2019 novel coronavirus–infected pneumonia. J. Med. Virol..

[13] Agrawal S., Orschler L., Lackner S. (2021). Long-term monitoring of SARS-CoV-2 RNA in wastewater of the Frankfurt metropolitan area in Southern Germany. Sci. Rep..

[14] Medema G., Been F., Heijnen L., Petterson S. (2020). Implementation of environmental surveillance for SARS-CoV-2 virus to support public health decisions: Opportunities and challenges. Curr. Opin. Environ. Sci. Health.

[15] Ahmed W., Angel N., Edson J., Bibby K., Bivins A., O’Brien J.W., Choi P.M., Kitajima M., Simpson S.L., Li J. (2020). First confirmed detection of SARS-CoV-2 in untreated wastewater in Australia: A proof of concept for the wastewater surveillance of COVID-19 in the community. Sci. Total Environ..

[16] Kitajima M., Ahmed W., Bibby K., Carducci A., Gerba C.P., Hamilton K.A., Haramoto E., Rose J.B. (2020). SARS-CoV-2 in wastewater: State of the knowledge and research needs. Sci. Total Environ..

[17] Prado T., Fumian T.M., Mannarino C.F., Resende P.C., Motta F.C., Eppinghaus A.L.F., Vale V.H.C.D., Braz R.M.S., Andrade J.D.S.R.D., Maranhão A.G. (2021). Wastewater-based epidemiology as a useful tool to track SARS-CoV-2 and support public health policies at municipal level in Brazil. Water Res..

[18] Thompson J.R., Nancharaiah Y.V., Gu X., Lee W.L., Rajal V.B., Haines M.B., Girones R., Ng L.C., Alm E.J., Wuertz S. (2020). Making waves: Wastewater surveillance of SARS-CoV-2 for population-based health management. Water Res..

[19] Ahmed W., Tscharke B., Bertsch P.M., Bibby K., Bivins A., Choi P., Clarke L., Dwyer J., Edson J., Nguyen T.M.H. (2020). SARS-CoV-2 RNA monitoring in wastewater as a potential early warning system for COVID-19 transmission in the community: A temporal case study. Sci. Total Environ..

[20] Medema G., Heijnen L., Elsinga G., Italiaander R., Brouwer A. (2020). Presence of SARS-Coronavirus-2 RNA in Sewage and Correlation with Reported COVID-19 Prevalence in the Early Stage of the Epidemic in The Netherlands. Environ. Sci. Technol. Lett..

[21] Hasan S.W., Ibrahim Y., Daou M., Kannout H., Jan N., Lopes A., Alsafar H., Yousef A.F. (2020). Detection and quantification of SARS-CoV-2 RNA in wastewater and treated effluents: Surveillance of COVID-19 epidemic in the United Arab Emirates. Sci. Total Environ..

[22] Hata A., Hara-Yamamura H., Meuchi Y., Imai S., Honda R. (2020). Detection of SARS-CoV-2 in wastewater in Japan during a COVID-19 outbreak. Sci. Total Environ..

[23] La Rosa G., Iaconelli M., Mancini P., Ferraro G.B., Veneri C., Bonadonna L., Lucentini L., Suffredini E. (2020). First detection of SARS-CoV-2 in untreated wastewaters in Italy. Sci. Total Environ..

[24] COVID19WBEC COVID-19 WBE Publication Map. https://www.covid19wbec.org/publication-map.

[25] Heijnen L., Elsinga G., de Graaf M., Molenkamp R., Koopmans M.P., Medema G. (2021). Droplet digital RT-PCR to detect SARS-CoV-2 signature mutations of variants of concern in wastewater. Sci. Total Environ..

[26] Yaniv K., Ozer E., Shagan M., Lakkakula S., Plotkin N., Bhandarkar N.S., Kushmaro A. (2021). Direct RT-qPCR assay for SARS-CoV-2 variants of concern (Alpha, B.1.1.7 and Beta, B.1.351) detection and quantification in wastewater. Environ. Res..

[27] Caduff L., Dreifuss D., Schindler T., Devaux A.J., Ganesanandamoorthy P., Kull A., Stachler E., Fernandez-Cassi X., Beerenwinkel N., Kohn T. (2021). Inferring Transmission Fitness Advantage of SARS-CoV-2 Variants of Concern in Wastewater Using Digital PCR. medRxiv.

[28] Yaniv K., Ozer E., Kushmaro A. (2021). SARS-CoV-2 Variants of Concern, Gamma (P.1) and Delta (B.1.617), Sensitive Detection and Quantification in Wastewater Employing Direct RT-QPCR. medRxiv.

[29] Baaijens J.A., Zulli A., Ott I.M., Petrone M.E., Alpert T., Fauver J.R., Kalinich C.C., Vogels C.B.F., Breban M.I., Duvallet C. (2021). Variant Abundance Estimation for SARS-CoV-2 in Wastewater Using RNA-Seq Quantification. medRxiv.

[30] Alygizakis N., Markou A.N., Rousis N.I., Galani A., Avgeris M., Adamopoulos P.G., Scorilas A., Lianidou E.S., Paraskevis D., Tsiodras S. (2020). Analytical methodologies for the detection of SARS-CoV-2 in wastewater: Protocols and future perspectives. TrAC Trends Anal. Chem..

[31] Gibson K., Schwab K., Spencer S., Borchardt M. (2012). Measuring and mitigating inhibition during quantitative real time PCR analysis of viral nucleic acid extracts from large-volume environmental water samples. Water Res..

[32] Bertels X., Demeyer P., Bogaert S.V.D., Boogaerts T., van Nuijs A.L., Delputte P., Lahousse L. (2022). Factors influencing SARS-CoV-2 RNA concentrations in wastewater up to the sampling stage: A systematic review. Sci. Total Environ..

[33] Li B., Deng A., Li K., Hu Y., Li Z., Shi Y., Xiong Q., Liu Z., Guo Q., Zou L. (2022). Viral infection and transmission in a large, well-traced outbreak caused by the SARS-CoV-2 Delta variant. Nat. Commun..

[34] Jahn K., Dreifuss D., Topolsky I., Kull A., Ganesanandamoorthy P., Fernandez-Cassi X., Bänziger C., Devaux A.J., Stachler E., Caduff L. (2021). Detection and Surveillance of SARS-CoV-2 Genomic Variants in Wastewater. medRxiv.

[35] Joshi M., Kumar M., Srivastava V., Kumar D., Rathore D., Pandit R., Joshi C.G. (2021). First Detection of SARS-CoV-2 Delta Variant (B.1.617.2) in the Wastewater of (Ahmedabad), India. medRxiv.

[36] Lin X., Glier M., Kuchinski K., Mierlo T.R.-V., McVea D., Tyson J.R., Prystajecky N., Ziels R.M. (2021). Assessing Multiplex Tiling PCR Sequencing Approaches for Detecting Genomic Variants of SARS-CoV-2 in Municipal Wastewater. medRxiv.

[37] Pérez-Cataluña A., Chiner-Oms Á., Cuevas-Ferrando E., Díaz-Reolid A., Falcó I., Randazzo W., Girón-Guzmán I., Allende A., Bracho M.A., Comas I. (2021). Detection of Genomic Variants of SARS-CoV-2 Circulating in Wastewater by High-Throughput Sequencing. medRxiv.

[38] Lee W.L., Gu X., Armas F., Chandra F., Chen H., Wu F., Leifels M., Xiao A., Desmond Chua F.J., Kwok G.W.C. (2021). Quantitative SARS-CoV-2 Tracking of Variants Delta, Delta plus, Kappa and Beta in Wastewater by Allele-Specific RT-QPCR. medRxiv.

[39] Peterson S.W., Lidder R., Daigle J., Wonitowy Q., Nagasawa A., Mulvey M.R., Mangat C.S. (2021). RT-QPCR Detection of SARS-CoV-2 Mutations S 69-70 Del, S N501Y and N D3L Associated with Variants of Concern in Canadian Wastewater Samples. medRxiv.

[40] Carcereny A., Martínez-Velázquez A., Bosch A., Allende A., Truchado P., Cascales J., Romalde J.L., Lois M., Polo D., Sánchez G. (2021). Monitoring Emergence of SARS-CoV-2 B.1.1.7 Variant through the Spanish National SARS-CoV-2 Wastewater Surveillance System (VATar COVID-19) from December 2020 to March 2021. medRxiv.

[41] Gering E., Colbert J., Schmedes S., Duncan G., Lopez J., Motes J., Weiss J., Azarian T., Tekin O., Blanton J. (2021). DdPCR Reveals SARS-CoV-2 Variants in Florida Wastewater. medRxiv.

[42] Corpuz M.V.A., Buonerba A., Vigliotta G., Zarra T., Ballesteros F., Campiglia P., Belgiorno V., Korshin G., Naddeo V. (2020). Viruses in wastewater: Occurrence, abundance and detection methods. Sci. Total Environ..

[43] Boogaerts T., Jacobs L., De Roeck N., Bogaert S.V.D., Aertgeerts B., Lahousse L., van Nuijs A.L., Delputte P. (2021). An alternative approach for bioanalytical assay optimization for wastewater-based epidemiology of SARS-CoV-2. Sci. Total Environ..

[44] Hokajärvi A.-M., Rytkönen A., Tiwari A., Kauppinen A., Oikarinen S., Lehto K.-M., Kankaanpää A., Gunnar T., Al-Hello H., Blomqvist S. (2021). The detection and stability of the SARS-CoV-2 RNA biomarkers in wastewater influent in Helsinki, Finland. Sci. Total Environ..

[45] Korukluoglu G., Kolukirik M., Bayrakdar F., Ozgumus G.G., Altas A.B., Cosgun Y., Ketre Kolukirik C.Z. (2021). 40 Minutes RT-QPCR Assay for Screening Spike N501Y and HV69-70del Mutations. bioRxiv.

[46] Centers for Disease Control and Prevention Research Use Only 2019-Novel Coronavirus (2019-nCoV) Real-time RT-PCR Primers and Probes. https://www.cdc.gov/coronavirus/2019-ncov/lab/rt-pcr-panel-primer-probes.html.

[47] Shu Y., McCauley J. (2017). GISAID: Global initiative on sharing all influenza data—From vision to reality. Eurosurveillance.

[48] Van Poelvoorde L.A.E., Gand M., Fraiture M.-A., De Keersmaecker S.C.J., Verhaegen B., Van Hoorde K., Cay A.B., Balmelle N., Herman P., Roosens N. (2021). Strategy to Develop and Evaluate a Multiplex RT-ddPCR in Response to SARS-CoV-2 Genomic Evolution. Curr. Issues Mol. Biol..

[49] Uhlig S., Frost K., Colson B., Simon K., Mäde D., Reiting R., Gowik P., Grohmann L. (2015). Validation of qualitative PCR methods on the basis of mathematical-statistical modelling of the probability of detection. Accredit. Qual. Assur..

[50] National Reference Laboratory (UZ Leuven & KU Leuven) (2022). Genomic Surveillance of SARS-CoV-2 in Belgium.

[51] Baker M. (2012). Digital PCR hits its stride. Nat. Methods.

[52] Dingle T., Sedlak R.H., Cook L., Jerome K.R. (2013). Tolerance of Droplet-Digital PCR vs. Real-Time Quantitative PCR to Inhibitory Substances. Clin. Chem..

[53] Suo T., Liu X., Feng J., Guo M., Hu W., Guo D., Ullah H., Yang Y., Zhang Q., Wang X. (2020). ddPCR: A more accurate tool for SARS-CoV-2 detection in low viral load specimens. Emerg. Microbes Infect..

[54] Klein D. (2002). Quantification using real-time PCR technology: Applications and limitations. Trends Mol. Med..

[55] Kuypers J., Jerome K.R. (2017). Applications of Digital PCR for Clinical Microbiology. J. Clin. Microbiol..

