# Supplementary material for: Optimization and Application of a Multiplex Digital PCR Assay for the Detection of SARS-CoV-2 Variants of Concern in Belgian Influent Wastewater
Source: Viruses. 2022 Mar 15;14(3):610. doi: 10.3390/v14030610 (PMC8953730; doi:10.3390/v14030610)
Supplement: Supplementary file 1 [file viruses-14-00610-s001.zip › viruses-1609838-supplementary.pdf]

Supplementary information

Optimization and application of a multiplex digital PCR assay for the detection of SARS-CoV-2 variants of concern in Belgian influent wastewater

Tim Boogaerts[1,*], Siel Van den Bogaert[2,*], Laura A.E. Van Poelvoorde[3], Diala El Masri[2], Naomi De Roeck[2], Nancy H. C. Roosens[3], Marie Lesenfants[4], Lies Lahousse[5], Koenraad Van Hoorde[6], Alexander L.N. van Nuijs[1,**], Peter Delputte[2,**]

1 Toxicological Centre, University of Antwerp, Universiteitsplein 1 2610 Wilrijk, Belgium.

2 Laboratory for Microbiology, Parasitology and Hygiene, University of Antwerp, Universiteitsplein 1 2610 Wilrijk, Belgium

3 Scientific directorate of Biological health risks, Service Transerversal activities in Applied Genomics, Sciensano, J. Wytsmanstraat 14, 1050, Brussels, Belgium

4 Scientific Directorate of Epidemiology and Public Health, Service Epidemiology of infectious diseases, Sciensano, J. Wytsmanstraat 14, 1050, Brussels, Belgium

5 Department of Bioanalysis, Ghent University, Ottergemsesteenweg 460, 9000 Ghent, Belgium

6 Scientific Directorate of Infectious diseases in humans, Service Foodborne pathogens, Sciensano, J. Wytsmanstraat 14, 1050, Brussels, Belgium

* joint first author

** joint senior author

Corresponding author: Tim.Boogaerts@uantwerpen.be

Supplementary information

Table S1 In-silico inclusivity and specificity of the different PCR targets

Assay	Name_probe	Sequence_probe	Inclusivity	FN	Target variant	Match target variant	Match target variant (#seq)	#Other variants (>50%)	#Other variants (>50%) (#seq)
1	Del69_70-Forward	TCAACTCAGGACTTGTTCTTACCT	98.39%	38 441	B.1.1.7	99.27%	558 458		
1	Del69_70-Reverse	GTTTGATAACCCTGTCCTACCA	98.52%	35 419	B.1.1.7	99.50%	558 458		
1	Del69_70-probe	TTCCATGCTATACATGTCTCTGGGA	74.19%	617 280	B.1.1.7	99.91%	558 458	58	589 314
2	B.1.1.7_Specific-Forward	GTTCTTACCTTTCTTTTCCAATGTTAC	99.14%	20 486	B.1.1.7	99.43%	558 458		
2	B.1.1.7_Specific-Reverse	CCCTGTCCTACCATTTAATGATGG	99.39%	14 521	B.1.1.7	99.60%	558 458		
2	B.1.1.7_Specific-Probe	TGGTTCCATGCTATCTCTGGGACC	24.06%	1 816 229	B.1.1.7	98.89%	558 458	35	19 858
3	N501YMutation-Forward	CATATGGTTTCCAACCCACTT	26.27%	1 763 377	B.1.1.7	99.85%	558 458	48	64 129
3	N501YMutation-Reverse	ACTTTCTTTTGAACTTCTACATGCACC	99.55%	10 749	B.1.1.7	99.62%	558 458		
3	N501YMutation-Probe	TGGTGTTGGTTACCAACCATACAGAG	99.42%	13 958	B.1.1.7	99.63%	558 458		
4	B.1.351_Specific-Forward	AGATTTGCCAATAGGTATTAACATC	99.41%	14 131	B.1.351	99.89%	5 694		
4	B.1.351_Specific-Reverse	GACTCCTGGTGATTCTTCTTCAG	96.87%	74 747	B.1.351	98.03%	5 694		
4	B.1.351_Specific-Probe	CTAGGTTTCAAACTTTACATAGAAGTT	0.29%	2 384 704	B.1.351	98.70%	231	3	5 842
5	B.1.617.2-F21989	GTTTATTACCACAAAAACAACAAAAG	73.22%	640 369	B.1.617.2	99.19%	61 612		
5	B.1.617.2-R22083	CACTTTTGAATATGTCTCTCAGCC	99.47%	12 790	B.1.617.2	99.61%	61 612		
5	S157	TGGATGGAAAGTGGAGTTTATTCTAGT	51.87%	1 151 056	B.1.617.2	97.74%	61 612	183	1 179 097

Table S2 In-silico specificity of the final dPCR target list on the different variants of concern (VOC) and variants of interest (VOI)

WHO Label	Data of designation	Lineage	First detection in country	Spike mutations of interest	Additional AA changes monitored	Evidence for impact on transmissibility	Evidence for impact on immunity	Evidence for impact on severity	S-N501Y	SΔ69/70 deletion	S-del L242-244L	S-del 156-157
Alpha	18-12-20	B.1.1.7	UK (Sep-2020)	N501Y, D614G, P681H	E484K & L452R	Yes	No	Yes	X	X		
Beta	18-12-20	B.1.351	South Africa (May-2020)	K417N, E484K, N501Y, D614G, A701V	L18F	Yes	Yes	Yes	X		X	
Gamma	11-01-20	P.1	Brazil (Nov-2020)	K417T, E484K, N501Y, D614G, H655Y	P681H	Yes	Yes	Yes	X			
Delta	11-05-21	B.1.617.2	India (Oct-2020)	L452R, T478K, D614G, P681R	K417N	Yes	Yes	Yes				X
Omicron	26-11-21	B.1.1.529	Multiple countries (Nov-2021)	A67V, Δ69-70, T95I, G142D, Δ143-145, N211I, Δ212, ins215EPE, G339D, S371L, S373P, S375F, K417N, N440K, G446S, S477N, T478K, E484A, Q493R, G496S, Q498R, N501Y, Y505H, T547K, D614G, H655Y, N679K, P681H, N764K, D796Y, N856K, Q954H, N969K, L981F		Yes	Yes		X	X		
-	22-04-21	B.1.620	Unclear	S477N, E484K, D614G, P681H			Yes			X	(X)	
Mu	30-08-21	B.1.621	Colombia	R346K, E484K, N501Y, D614G, P681H		Yes	Yes		X			
Lambda	14-06-21	C.37	Peru	L452Q, F490S, D614G			Yes					
Eta	17-03-21	B.1.525	Multiple countries	E484K, D614G, Q677H			Yes			X		
Iota	24-03-21	B.1.526	US	E484K, D614G, A701V			Yes					
Kappa	04-04-21	B.1.617.1	India	L452R, E484Q, D614G, P681R		Yes	Yes					

Table S1 Assessment of the LOD95% of the different variant specific primer sets. For each concentration level, the positive detection rate (%) among replicates was given in italics together with the mean copy number (copies/µL ± standard deviation) as observed with the dPCR method.

	**Del69/70**	**N501Y**	**B.1.1.7**	**B.1.351**	**B.1.617.2**
**L1**	*100%*<br>221.1 ± 9.6	*100%*<br>92.4 ± 1.8	*100%*<br>131.8 ± 5.9	*100%*<br>14.0 ± 5.6	*100%*<br>369.8 ± 22.3
**L2**	*100%*<br>112.7 ± 1.8	*100%*<br>41.8 ± 0.7	*100%*<br>62.9 ± 7.1	*100%*<br>7.7 ± 0.4	*100%*<br>181.7 ± 5.1
**L3**	*100%*<br>39.8 ± 1.0	*100%*<br>13.2 ± 1.2	*100%*<br>26.2 ± 1.5	*100%*<br>3.4 ± 1.8	*100%*<br>73.2 ± 5.4
**L4**	*100%*<br>20.3 ± 2.0	*100%*<br>6.7 ± 1.1	*100%*<br>11.8 ± 0.7	*100%*<br>1.3 ± 0.1	*100%*<br>37.7 ± 1.6
**L5**	*100%*<br>10.9 ± 1.1	*100%*<br>3.6 ± 0.4	*100%*<br>5.4 ± 0.5	*100%*<br>0.8 ± 0.4	*100%*<br>16.2 ± 3.5
**L6**	*100%*<br>4.3 ± 0.7	*100%*<br>1.2 ± 0.3	*100%*<br>2.6 ± 0.9	*100%*<br>0.4 ± 0.1	*100%*<br>8.7 ± 0.3
**L7**	*100%*<br>0.5 ± 0.2	*66%*<br>0.1 ± 0.1	*100%*<br>0.4 ± 0.2	*33%*<br>0.03 ± 0.06	*33%*<br>0.4 ± 0.8
**LOD95% [95% confidence interval]**	<0.5	0.3 [0.1; 1.7]	<0.4	0.4 [0.2; 1.3]	2.9 [0.4; 20.5]
